# Crystal Structure of *Proteus mirabilis* Lipase, a Novel Lipase from the *Proteus/*Psychrophilic Subfamily of Lipase Family I.1

**DOI:** 10.1371/journal.pone.0052890

**Published:** 2012-12-26

**Authors:** Tyler P. Korman, James U. Bowie

**Affiliations:** 1 Department of Chemistry and Biochemisty, University of California Los Angeles, Los Angeles, California, United States of America; 2 UCLA-DOE Institute of Genomics and Proteomics, University of California Los Angeles, Los Angeles, California, United States of America; 3 Molecular Biology Institute, University of California Los Angeles, Los Angeles, California, United States of America; Monash University, Australia

## Abstract

Bacterial lipases from family I.1 and I.2 catalyze the hydrolysis of triacylglycerol between 25–45°C and are used extensively as biocatalysts. The lipase from *Proteus mirabilis* belongs to the *Proteus*/psychrophilic subfamily of lipase family I.1 and is a promising catalyst for biodiesel production because it can tolerate high amounts of water in the reaction. Here we present the crystal structure of the *Proteus mirabilis* lipase, a member of the *Proteus/*psychrophilic subfamily of I.1lipases. The structure of the *Proteus mirabilis* lipase was solved in the absence and presence of a bound phosphonate inhibitor. Unexpectedly, both the apo and inhibitor bound forms of *P. mirabilis* lipase were found to be in a closed conformation. The structure reveals a unique oxyanion hole and a wide active site that is solvent accessible even in the closed conformation. A distinct mechanism for Ca^2+^ coordination may explain how these lipases can fold without specific chaperones.

## Introduction

The ability of an organism to convert triacylglycerol to free fatty acids is mediated by lipases (triacylglycerol hydrolase, EC 3.1.1.3) [1]–[3]. Although naturally occurring oils are the primary lipase substrate, synthetic esters are also hydrolyzed by lipases, often stereospecifically. Additionally, in low water environments lipases will catalyze the synthesis of esters from an alcohol and carboxylic acid (esterification) or an alcohol and another ester (transesterification) [4]. As a result, lipases have widespread biotechnological applications in detergents, in the production of pharmaceuticals, and in the synthesis of esters for use in the food and energy industries [1]. The great biotechnological potential of lipases has prompted many efforts to discover new lipases or generate modified lipases with improved properties for specific applications [5].

Of particular biotechnological interest are lipases from microbial sources. Many microbial lipases have been characterized and are used industrially [5]. Of the bacterial lipases, those from family I.1 and I.2 are perhaps the best studied [6]. Lipases from family I.1 and I.2 share greater than 40% sequence identity and are nearly exclusively found in *Pseudomonas* and *Proteus* species. Despite a high degree of sequence similarity, family I.1 and I.2 lipases display diverse properties such as enantioselectivity, thermostability, and solvent tolerance that are unique to each lipase. Recently, lipases from various *Proteus* species have garnered interest as biocatalysts due to their high activity, tolerance to organic solvents, and ability to express solubly in *E. coli* in a chaperone independent manner [7]–[10]. The lipase from *Proteus mirabilis* is a family I.1 lipase (100% identical to *Proteus sp. k107* lipase [9]) that has potential for practical applications (hereafter referred to as PML). For example, PML was found to be an excellent catalyst for biodiesel production at ambient temperature [9]. Subsequent studies showed that PML also catalyzes the enantiospecific hydrolysis of chiral esters and can be re-engineered for improved catalysis [11], [12].

Structures of PML homologs from *Pseudomonas aeruginosa* (PAL) [13], *Burkholderia cepacia* (BCL) [14], [15], and *Burkholderia glumae* (BGL) [16] have been solved. PAL is a I.1 family lipase and BCL and BGL are I.2 family lipases. PAL, BCL, and BGL share a common α/β hydrolase fold [17], Asp-His-Ser catalytic triad, and preformed oxyanion hole necessary for catalysis [6], [17], [18]. They all possess a lid domain that is thought to close over the active site in aqueous solution, but open when in contact with an oil surface, leading to interfacial activation of the enzymes [19]. The lid domain is in a different conformation in PAL and BCL compared to BGL, providing insight into the changes that occur upon interfacial activation. While structures of PAL, BCL, and BGL provide a general rationale for lid opening (interfacial activation), novel structures are needed to elucidate the specific features governing the molecular basis of catalysis and enantioselectivity catalyzed by individual lipases. To date, only three unique lipases from families I.1 and I.2 have known structures. PAL is the only family I.1 lipase solved. Here we present crystal structures of PML in the absence and presence of diethyl phosphonate, a potent lipase inhibitor. Both PML structures have been solved in the closed conformation and provide further insight into catalysis by family I.1 and I.2 lipases.

## Materials and Methods

### Cloning, Expression, and Purification

The lipase gene from *Proteus mirabilis* was amplified from genomic DNA (ATCC12453D) by the polymerase chain reaction (PCR). The PCR product was inserted into a pET28a vector to generate plasmid pTK05, which encodes the *P. mirabilis* lipase with an N-terminal hexahistidine tag.

pTK05/BL21Gold(DE3) was grown in 2 L of LB media containing 50 µg/mL kanamycin at 37°C to OD_600_ of 0.6. Protein expression was induced with 0.5 mM IPTG and the culture incubated at 16°C for an additional 16 hours. Cells were harvested by centrifugation for 20 min at 4000 rpm in a Sorvall GS3 rotor, resuspended in 100 mL of Buffer A (50 mM Tris-Cl pH 7.5, 0.3 M NaCl, 10 mM imidazole) and lysed by sonication at 4°C. The lysate as centrifuged at 13000 rpm in a Sorvall SS-34 rotor for 40 min and the supernatant was applied to a 5 mL column of Ni-NTA (Qiagen). The column was washed with 20 mL Buffer A followed by elution of PML with Buffer A containing 250 mM imidazole to yield ∼80 mg of protein that was >95% pure. The recombinant PML was then dialyzed overnight into 20 mM Tris-Cl pH 7.5 containing 100 mM NaCl and flash cooled as droplets in liquid N_2_ prior to storage at −80°C.

### Crystallization of Native and Inhibitor Bound PML

To generate the PML-inhibitor complex, 10 mL of 10 mg/mL PML was treated with 2 mM paraoxon (DEP) and 0.6% Triton X100 for 18 hours at 25°C. The DEP inhibited enzyme was then bound to a 1 mL Ni-NTA column and washed with 20 mL of 20 mM Tris-Cl pH 7.5, 0.2 M NaCl to remove Triton X100 and unreacted inhibitor. Inhibited protein was then eluted with 5 mL buffer containing 250 mM imidazole, dialyzed into 20 mM Tris-Cl, pH 7.5, 0.1 M NaCl, and concentrated to 10 mg/mL. Inhibited protein was then stored as the native protein prior to crystallization.

Crystals of native or DEP-inhibited PML were formed by vapor diffusion as hanging drops over 500 µL of well solution at 25°C. Drops were generated by mixing 2 µL of protein at 9 mg/mL with 2 µL well solution. Large crystals of the native PML formed within one week from two well solution conditions: 0.1 M Hepes pH 7.5, 70% MPD or 0.1 M Sodium Citrate pH 5.6, 20% isopropanol, 20% PEG4K, 5% glycerol. Crystals of DEP-inhibited PML were obtained from 0.1 M bis-tris propane pH 6.5, 15% PEG2K MME. All crystals of DEP inhibited PML in PEG2K MME were twinned (see below). Prior to data collection, native crystals from were soaked in crystallization solution plus 15% glycerol and flash frozen in liquid nitrogen.

### Structure Determination, and Refinement

All data were collected in house on a Rigaku FRE+ x-ray generator equipped with an ADSC Quantum 4 CCD detector at 100 K. Diffraction images were indexed, integrated, and scaled with Denzo and Scalepack. Initial phases for native PML were determined by molecular replacement using PHASER [20] in CCP4i [21]. The *Pseudomonas aeruginosa* lipase (41% identity; PDBID 1EX9) was used as the search model with lid helix (PAL residues 112 to 154) omitted. One round of refinement with Refmac5 [22] gave initial R and R_free_ values of 40% and 45% over the resolution range 50–2.0 Å. The lid helices were added and the resulting model was refined via iterative rounds of model building and refinement in COOT [23] and Refmac5. Waters were added using COOT along with isopropanol, the defined portion of PEG4k molecules, and Ca^2+^. The final round of energy minimization and B factor refinement gave final R and R_free_ values of 16.7% and 19.4% for the native PML.

Phases for DEP-inhibited PML were determined as described above using the native PML lipase as the search model. For the DEP inhibited structure, the *R* and *R*_free_ values did not decrease below 32% and 37%. The data was checked for twinning using the Padilla-Yeates Algorithm [24] and the twin fraction and operator determined using PHENIX [25]. The data were corrected for twinning by using DETWIN [26] in CCP4i with the twinning operator h, -h-k, -l and a twin fraction of 0.327, resulting in a decrease in R and *R*_free_. At this point, waters were added using COOT along with the DEP molecule, the defined portion of PEG4k molecule, and one Ca^2+^ ion followed by iterative rounds of refinement with Refmac5. The final R and *R*_free_ for the DEP inhibited structure is 19.5% and 24.9% respectively between 32.8–2.2 Å. A 2*Fo-Fc* simulated annealing omit map around the covalently bound DEP was generated using CNS [27] to unambiguously confirm the bound ligand (Figure S1). The crystal parameters and refinement statistics are given in Table S1 in Supplementary Materials. All residues could be modeled in both structures except residues 149 and 150 which are disordered. The backbone torsion angles of L13 are outside the accepted Ramachandran limits in both structures. However, the density in this region is very clear and cannot be modeled in another conformation (see below). Besides the presence of a covalently bound inihibitor, there is relatively little difference between the DEP inhibited and PEG/Isopropanol bound PML structures which display an RMSD of 0.23 Å for all Cα atoms. SwissPDB Viewer [28] was used to overlay structures and calculate RMSD. CLUSTALW [29] was used to generate alignments and calculate sequence identity. The native and DEP inhibited structures were deposited as PDBID 4GW3 and 4GXN respectively.

## Results and Discussion

### Overall Structure

**Figure 1:**
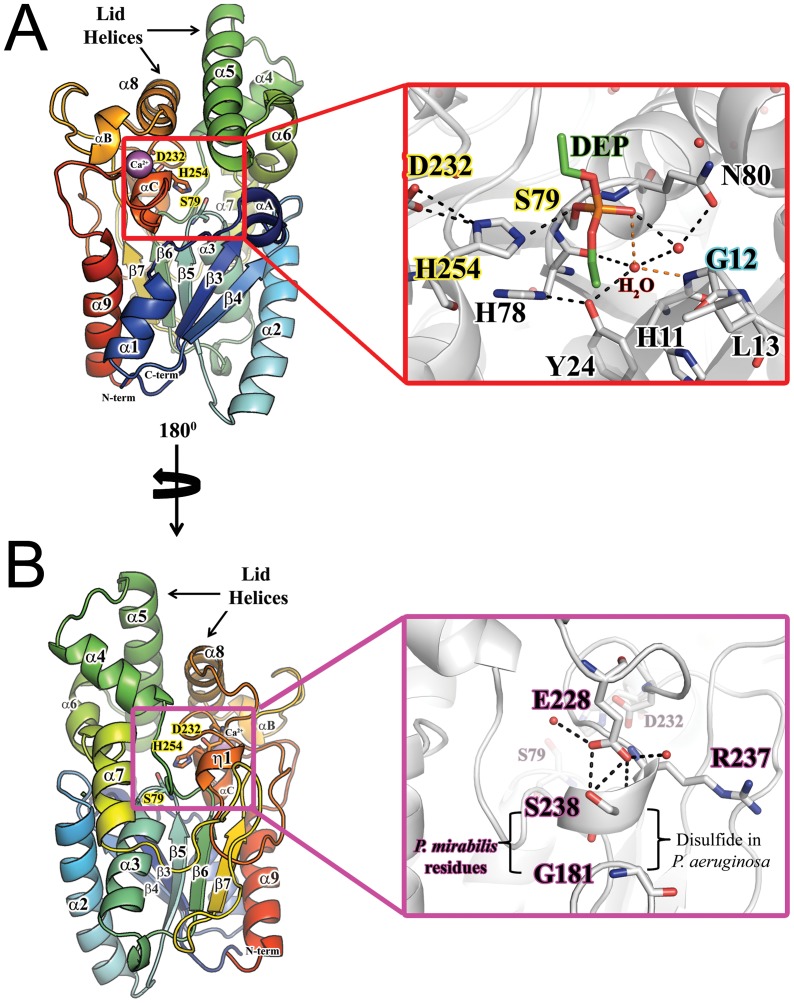
Structure of *P. mirabilis* lipase. **A**) Secondary structure elements of *P. mirabilis* lipase from lipase family I.1. β-sheets are numbered according to the corresponding β-sheet in the canonical α/β hydrolase fold. The cartoon is colored according to the sequence position, ranging from the N-terminus in blue to the C-terminus red. The active site residues are highlighted with yellow and shown as sticks. The bound Ca^2+^ is shown as a purple sphere. The inset box shows the PML active site with inhibitor DEP covalently bound to S79. The hydrogen bonds stabilizing the oxyanion are shown as orange dashes. The backbone amide of G12 (labeled with blue outline) has replaced L13 as the potential hydrogen bond donor helping to stabilize the oxyanion in the transition state. Other potential activating and stabilizing interactions are shown as black dashes. **B**) A 180° rotation of the orientation shown in (**A**) to expose the location of absent disulfide bond found in the PAL homolog. The region is outlined with a purple box and expanded in the inset. In PML, hydrogen bonds (black dashes) from the conserved E228 help stabilize helix η1 in the absence of a disulfide bond.

The structure of PML lipase is shown in Figure 1. We have numbered the helices and strands as described for PAL in reference [13]. Helices unique to PML compared to PAL are labeled with a letter while 3_10_ helices are labeled as η. The PML structure closely resembles the structure of the family I.1 PAL (1EX9, 42% sequence ID, 0.87 Å), as well as family I.2 lipases BGL (1CVL, 38% sequence ID, 1.05 Å) and BCL (1OIL, 38% sequence ID, 0.98 Å). Like the other family I.1 and I.2 lipases, PML adopts an α/β hydrolase fold with a central β-sheet core of 5 parallel β-strands and the first two βstrands are missing compared to the canonical α/β hydrolase fold [17]. The central core domains (excluding residues 214–236 in BGL and BCL and helix α5 in BCL and PAL) of all family I.1 and I.2 lipase structures overlay very well with an overall RMSD on Cα atoms of 0.87 Å, 0.98 Å, and 1.05 Å for PAL, BCL, and BGL respectively. In PML, the β-sheet core is surrounded by α helices α1, α2, α3, α7 and α9 while helices α4, α5, and α6 border the active site and help define the active site pocket (Figure 1A). Helix αA replaces a small antiparallel β-sheet between β3 and α1 that is found in PAL and PGL.

PML contains a typical hydrolase Ser-His-Asp catalytic triad with the catalytic serine, S79, part of a G-X-S-X-G motif between α3 and β5. In the DEP inhibited PML structure, the small phosphonate inhibitor is covalently bound to the Oγ of S79, mimicking the tetrahedral intermediate that would form during hydrolysis of triglycerides (Figure 1A and S3). There are no significant differences in the overall structure or the active site of PML upon covalent modification of S79 with DEP.

A major difference between PML and other I.1 and I.2 lipases of known structure is a conserved disulfide bond [13], [30] (Figure 1B) between residues 181 and 238 (PML numbering) that is lacking in PML. The disulfide bond serves to stabilize and orient helix α10 (equivalent to η1) so that D232 is placed appropriately in the active site to activate H254. Sequence comparison suggests that the homologous *Proteus* lipases (*P. vulgaris* [31] and *Proteus sp*. SW1 [7]) as well as the psychrophilic lipase from *Pseudomonas fragi* [32], also lack the disulfide bond (Figure S2). In PML, the disulfide bond is replaced by an H-bonding interaction between E228 to the side chain and backbone amide of S238. Although S238 is not conserved, E228 is conserved in *P. vulgaris* and *Proteus sp*. SW1 lipases and is shifted to E227 (PML numbering) in *P. fragi*, *and P. fluorescenes* lipases suggesting that the H-bond is a common strategy to stabilize helix η1 in the absence of a disulfide bond. It is tempting to speculate that the replacement of the disulfide bond may enable more conformational flexibility for folding or catalysis at low temperatures (for the psychrophilic lipases).

**Figure 2:**
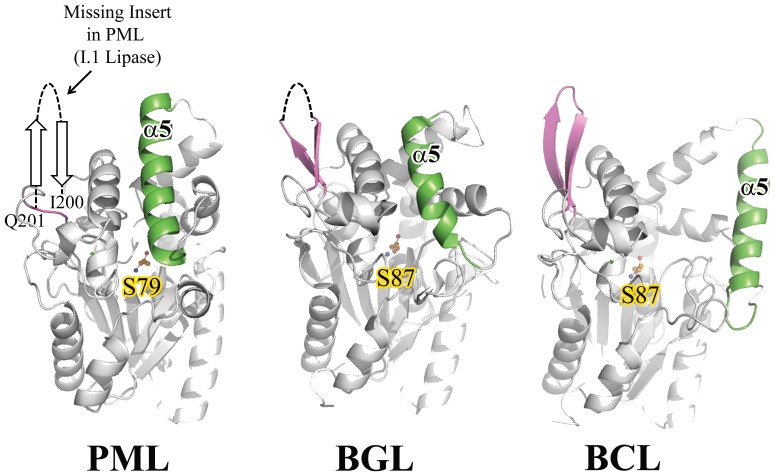
Comparison of the distinguishing β-turn-β structure that differentiates family I.1 lipases from family I.2. The position of the absent insertion in PML is shown. The ∼14 residue insertion in BGL and BCL (family I.2 lipases) is colored purple. As reference, lid helix α5and the catalytic serine are shown and colored green and orange respectively.

**Figure 3:**
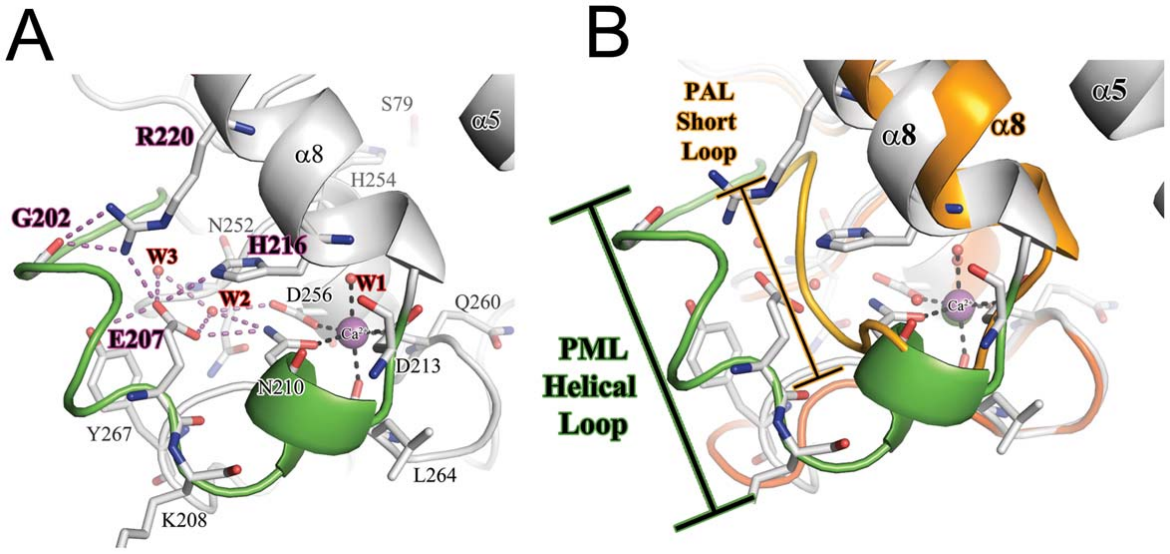
The structure of Ca^2+^ binding site and supporting loop region in PML. **A**) A detailed view of the C^2+^ binding site. The Ca^2+^ ion is shown as a purple sphere. The coordination of residues to Ca^2+^ are shown as black dashes. The intricate H-bonding network connecting the loop to lid helix α8 are shown as purple dashes. The helical loop region unique to *P. mirabilis* and other *Proteus*/psychrophilic subfamily I.1 lipases is shown in green. **B**) Comparison of the loop region in *P. mirabilis* (PML; green) and *P. aeruginosa* (PAL) orange. The loop that precedes the Ca^2+^ binding site is 5 residues longer in PML compared to PAL.

Another distinguishing feature between family I.1 and I.2 lipases is an insertion of 14 residues between residue I200 and Q201 (PML numbering) in family I.2 lipases that form an anti-parallel β sheet that precedes αB and α8 (Figure 2 and Figure S2). Likewise, it is missing in the PML lipase. However, the loop preceding αB adopts a helical conformation in PML and is 5 amino acids longer than the same region in PAL (Figure 3). In PML, this longer helical loop allows E207 to orient towards helix α8 and participate in an intricate H-bonding network with residues from α8 that may help stabilize both α8 and the Ca^2+^ binding site (see below).

### The Ca^2+^ Binding Site

A Ca^2+^ binding site located near the end of α8 is a conserved feature of family I.1 and I.2 lipases and has been proposed to play a role in catalysis and stability [33], [34]. Because the bound Ca^2+^ is coordinated to residues located on or near the helix containing the catalytic H254, it is likely that binding of Ca^2+^ is necessary to orient H254 properly to activate the nucleophile S79. As shown in Figure 3A, the Ca^2+^ ion in PML is found octahedrally coordinated to five main-chain or side-chain atoms and a single water. In PML, the Ca^2+^ is coordinated by atoms from the side chains of N210, D213, D256, the main-chain carbonyl oxygens from Q260 and L264, and a water molecule. A *cis*-peptide bond between Q260 and V261of PML is a conserved feature found in family I.1 and I.2 lipases [13], [15]. Additionally, PML contains an Asp residue, D253, that precedes the catalytic histidine H254 and hydrogen bonds with a conserved water molecule coordinated to the bound Ca^2+^. By sequence comparison, the *Proteus* sub-family of I.1 lipases contain an Asp at position 253 while mesophilic homologs contain an Asn. In the other I.1 and I.2 family lipases, the Ca^2+^ is coordinated by two water molecules, but in PML one of the waters is replaced by the side-chain of N210 in PML (Figure 3A). Although N210 is well conserved in family I.1 and I.2 lipases, only PML has N210 coordinated to the Ca^2+^. The positioning of N210 is accomplished by the presence of a structured helical loop mentioned above (residues 197–209 in PML) that precedes αB (Figure 3B). This structured loop is unique to the PML structure and is held in a defined conformation by an intricate H-bonding network that includes E207, H216, R220, the main-chain carbonyl oxygens from Q201 and G202 (*cis*-peptide bonded), and two water molecules. E207, H216, and R220, are well conserved in *Proteus* lipases (*P. vulgaris* 80%ID, *Proteus sp.* SW1 79%ID) as well as psychrophilic lipases from *Pseudomonas fragi* and *Pseudomonas fluorescens*, suggesting this unique loop is a common feature of these lipases (Figure S2).

Interestingly, the *Proteus* lipases and those from *Pseudomonas fragi* are amongst the few examples of lipases that fold in a chaperone independent manner [7], [8], [10]. The structure of PGL in complex with its cognate foldase in addition to recent protease protection data shows that there are many contacts made between the lipase and chaperone in this region, suggesting that this region near the Ca^2+^ binding site needs to be stabilized to ensure proper folding [35], [36].

### The Active Site Pocket

**Figure 4:**
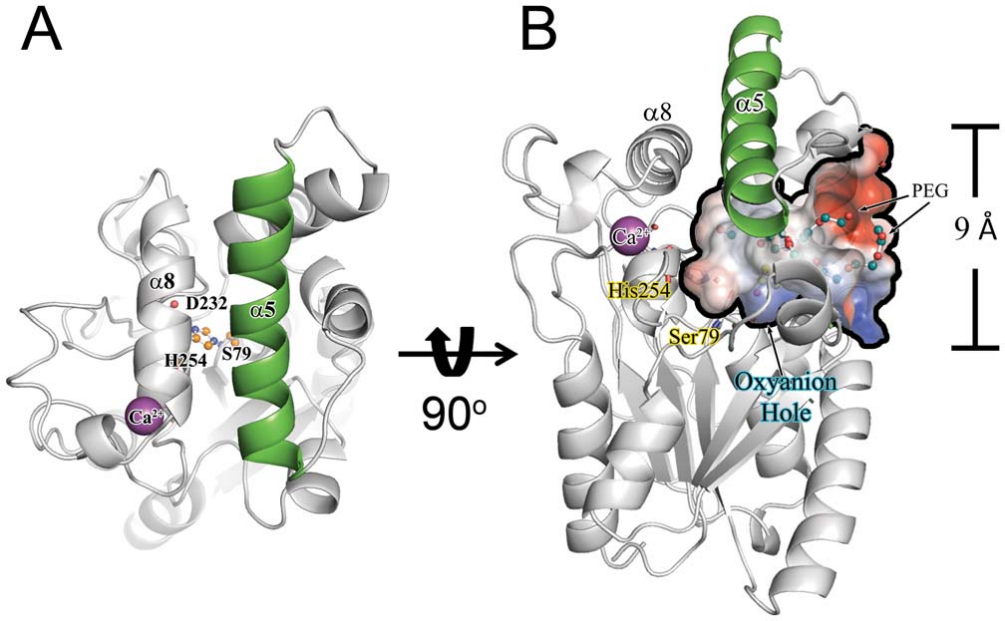
Water accessibility of the PML active site in the closed conformation. **A**) PML lid helix α5 (green) precludes access to the catalytic triad (orange ball and stick) suggesting PML is in a “closed” conformation. (**B**) Despite the location of α5, an ∼9 Å hole is present in PML that can accommodate at least 2 PEG molecules (blue ball and stick) seen in the structure.

The placement of the lid helices α5 and α8 suggest that PML is in a “closed” conformation with the position of α5 preventing access of long-chain triacylglycerols to the active site (Figure 4A). The position of α5 is similar to that seen in the closed conformation of BGL [16], [37] and in stark contrast to the position of the lid seen in the open conformation of PAL [13] that has an inhibitor bound (Figure S3). The closed conformation of lipases may be a strategy to prevent aggregation due to the interaction of hydrophobic surfaces in the absence of an oil/water interface [19]. Surprisingly, in both the DEP inhibited and apo structures of PML, the positively charged loop between α5 and α6 adopts a conformation that opens an ∼9Å diameter hole in PML (Figure 4B). The hole allows 2 PEG molecules from the crystallization solvent to be accommodated in a wide pocket that extends to S79 and exposes the active site of PML to solvent, even in the “closed” conformation. The presence of such a solvent exposed active site in the absence of an interface is in stark contrast to the closed conformation seen in PGL and suggests there may be an alternative mechanism of lid opening in the *Proteus*/psychrophilic family of lipases. Aiding in the exposure of the pocket is the presence and placement of αA (residues 15–20; Figure 1A; S2), which replaces a turn motif that is present in the PAL structure (D21–Y29). Assuming α5 moves to the same position seen in PAL upon exposure to a hydrophobic interface, αA would either have to move or α5 would have to take a different path upon “opening” (Figure S3). However, αA shows significant sequence diversity even among close homologs (>79%ID overall) suggesting the solvent accessibility and or specific mode of lid opening may be unique to each lipase.

As expected, the substrate binding pocket in PML is mostly hydrophobic. However, there is a relatively low conservation of the amphipathic lid (α5) and pocket residues of PML compared to PAL, BGL and BCL. The low conservation of pocket residues may contribute to observed differences in the stereo- and regio-specificity between family I.1 and I.2 lipases [38]. The PML pocket is lined by hydrophobic residues L13, F16, F47, V132, F136, I139, I140, F143, A153, A156, L157, L160, M199, L222, L234, and I255. Of these residues, only the side-chain of L234 moves appreciably upon binding of the covalent inhibitor DEP, adopting a similar conformation to L231 in PAL which also has a covalently bound inhibitor but is in an “open” conformation. In comparison to PAL, which opens to accommodate the bound octanoyl inhibitor, it is surprising that, not only does PML remain closed when DEP is bound, but that DEP can be accommodated in the pocket without significant movement of side chains and secondary structural elements. While access of DEP to the active site can be explained by the hole exposing the active site to solvent described above, the ability of DEP to covalently modify the active site S79, mimicking the oxyanion intermediate, appears to be possible due to the unique active site architecture of PML.

### The Oxyanion Hole

**Figure 5:**
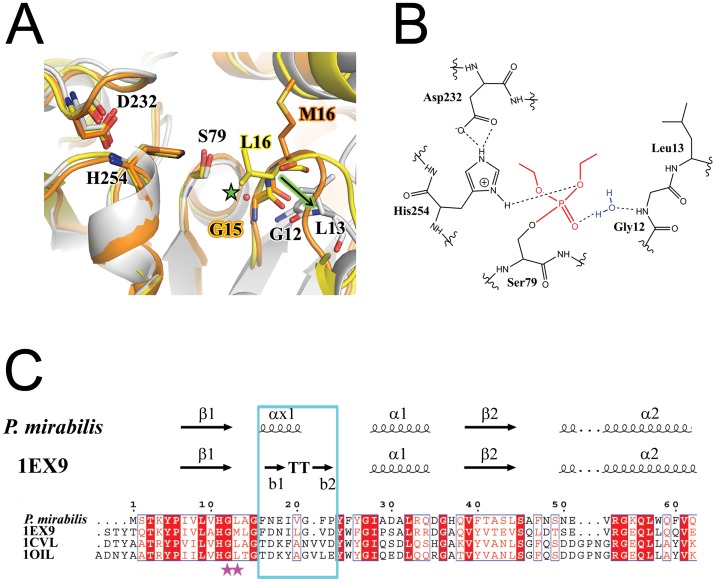
The oxyanion hole. **A**) Comparison of the loop stabilizing the oxyanion hole in PML (grey), PAL (orange) and BGL (yellow). The arrow (green) highlights the large movement of L13 in the loop (equivalent to M16 or L16 in PAL and BGL respectively) compared to PAL and BGL. The green star highlights the location of the oxyanion hole and the water that substitutes for the backbone amide seen in the other lipases with a preformed oxyanion hole. **B**) Schematic of the new water mediated stabilization of the oxanion hole found in PML due to a shift of the loop region seen in (**A**). **C**) Sequence comparision of the loop region in PML and other family I.1 and I.2 lipases of known structure. The loop region, highlighted in the blue box, is one residue shorter in family I.1 lipases compared to I.2 lipases.

One of the most surprising and interesting deviations of the PML structure(s) from the other known I.1 and I.2 lipases is the location and orientation of the oxyanion hole. As shown in Figure 5, S79 appears to be in a “catalytically competent” conformation in PML even in the absence of inhibitor with the Oγ of S79 pointed away from the Nε2 of H254 [16]. However, the oxyanion hole is markedly different in PML compared to PAL, BGL, or BCL. The observed differences in the PML oxyanion hole suggest that either the oxyanion hole is 1) unique in PML and much more open than in other I.1 and I.2 lipases of known structure or 2) *not* pre-formed as suggested for BGL which is also in the closed conformation but whose oxyanion hole residues occupy the same position seen in BCL and PAL which are both open structures. In either case, the alteration of the oxyanion hole represents a stark contrast to the assumptions made about catalysis in family I.1 and I.2 lipases.

To pinpoint the differences in the oxyanion holes, we compared the DEP inhibited PML with the structure of PAL which also has an inhibitor bound (Figure 5). This comparison should help to pinpoint key residues involved in the observed change of the oxyanion hole. In PAL, the oxyanion hole is composed of the backbone amide of H83 and of M16 which is part of the tetrapeptide motif (ie Gly-Hyd-X-Gly). Importantly, in PAL the oxyanion hole is devoid of water molecules. If the oxyanion hole was the same in PML, it should be composed solely of the backbone amides from Q80 and L13 (the tetrapeptide motif is 12-G-L-A-G-15). However, while the tetrapeptide motif is conserved in PML, in the DEP inhibited structure the phosphonate is stabilized by an oxyanion hole composed of the backbone amide of Q80 and two water molecules. One of the water molecules is bonded to the backbone amides of G12, and Y24, the backbone carbonyl of H78, and the oxyanion from the phosphonate inhibitor. The other water is bonded to the first water, the side chain of Q80, and the oxyanion. The presence of the water bound to G12 and the position of the loop bearing the tetrapeptide motif is unique to PML (Figure 5A, B).

The drastically different oxyanion hole seen in PML occurs due to a large shift of the tetrapeptide motif compared to PAL [13] (Figure 5A). Specifically, L13 is shifted 4.4 Å from the location of the equivalent M16 in PAL. It has been proposed that the oxyanion hole is stabilized by the interaction of the tetrapeptide motif with a conserved arginine. The tetrapeptide motif (12-G-L-A-G-15) and the arginine (R53) are conserved in PML, although R53 does not interact with the tetrapeptide motif. Instead, in PML R53 is rotated away from the oxyanion hole and the tetrapeptide motif (Fig. 1D, 2B). Perhaps more telling is the length of the loop between the conserved residues G12 and Y24. This region contains the tetrapeptide motif followed by αA, a short two turn helix that replaces a turn motif seen in PAL (BCL also contains a short helix in this position as well). The length of the peptide between G12 and Y24 is one residue shorter in PML compared to PAL (and PGL whose oxanion hole is pre-formed in the closed conformation), perhaps causing the large shift in the tetrapeptide motif (Figure 5C). When hydrolytic activity is assayed with the synthetic substrate p-nitrophenyl palmitate, the activity of PML is nearly four times less than BCL (not shown). It is possible that the lack of a completely preformed oxyanion hole compromises PML activity. This one residue deletion is conserved in the lipases from other *Proteus* species as well as *Pseudomonas fragi* and *Pseudomonas fluroescens*. It would be interesting to see whether the tetrapeptide motif remains in the same conformation in the open conformation.

The observed differences in the PML oxyanion hole suggests that the oxyanion hole is unique in PML and much more open than in other I.1 and I.2 lipases of known structure. It is also possible that the oxyanion hole is *not* pre-formed in the closed structure of PML. It is thought, however, that the oxyanion hole should not change in the open and closed conformations as the oxyanion hole in the closed PGL structure is in the same conformation as in the open PCL and PAL structures.

## Conclusions

The crystal structure of PML provides the first structure of a lipase from the *Proteus* I.1 sub-family that include psychrophilic lipases. While many of the global features of I.1 and I.2 lipases are conserved, there are some unique features of the Proteus I.1 family. We see changes in the way the Ca^2+^ is coordinated, which may be important for allowing these lipases to fold without chaperones as this region forms intimate contacts with specific foldases. We also see remarkable differences in the active site pocket as it is much more exposed and solvent accessible than the active site seen in other lipases of known structure, even though our PML structure otherwise appears to be in a closed conformation. It is possible that different substrates can access the active site by distinct mechanisms either in the closed or open conformation. Finally, the oxyanion hole is formed in a unique way in the PML structure compared to other lipases. Our results reveal new and unexpected diversity in the lipase enzyme family.

## Supporting Information

Figure S1 ***2Fo-Fc*** **sa-omit map contoured at 1 σ highlighting the covalently bound diethyl-phosphonate (DEP) inhibitor in the active site.**
(TIF)

Figure S2 **Multiple sequence alignment of psychrophilic and mesophilic lipases from lipase family I.1 and I.2.** The secondary structure elements are numbered as in Figure 1. Orange squares, catalytic triad residues. Ca2+ binding site, blue ovals. Disulfide bond, black triangle.
(TIF)

Figure S3 **Comparison of the open and closed conformations seen in family I.1 and I.2 lipases.** **A**) Overlay of closed PML (blue), closed *B. glumae* (PDBID:1CVL), and open *P. aeruginosa* (PDBID:1EX9) lipases. The helix αA that would need to move upon lid opening is highlighted with a red arrow. **B**) Surface representation of the solvent accessibility of the active site pocket of *B. glumae* (closed) and *P. aeruginosa* (open). The active site of *B. glumae* lipase is marginally accessible in the closed conformation. The open conformation of *P. aeruginosa* lipase shows a hydrophobic cleft that can accommodate a triglyceride analog (orange ball and stick) without steric clashes.
(TIF)

Table S1 **Crystallization, data collection and refinement statistics for the crystal structures of the apo and DEP inhibited** ***P. mirabilis*** **lipase.**
(DOC)
