# Supplementary figures and images for: Crystal Structure of Proteus mirabilis Lipase, a Novel Lipase from the Proteus/Psychrophilic Subfamily of Lipase Family I.1
Source: PLoS One. 2012 Dec 26;7(12):e52890. doi: 10.1371/journal.pone.0052890 (PMC3530535; doi:10.1371/journal.pone.0052890)

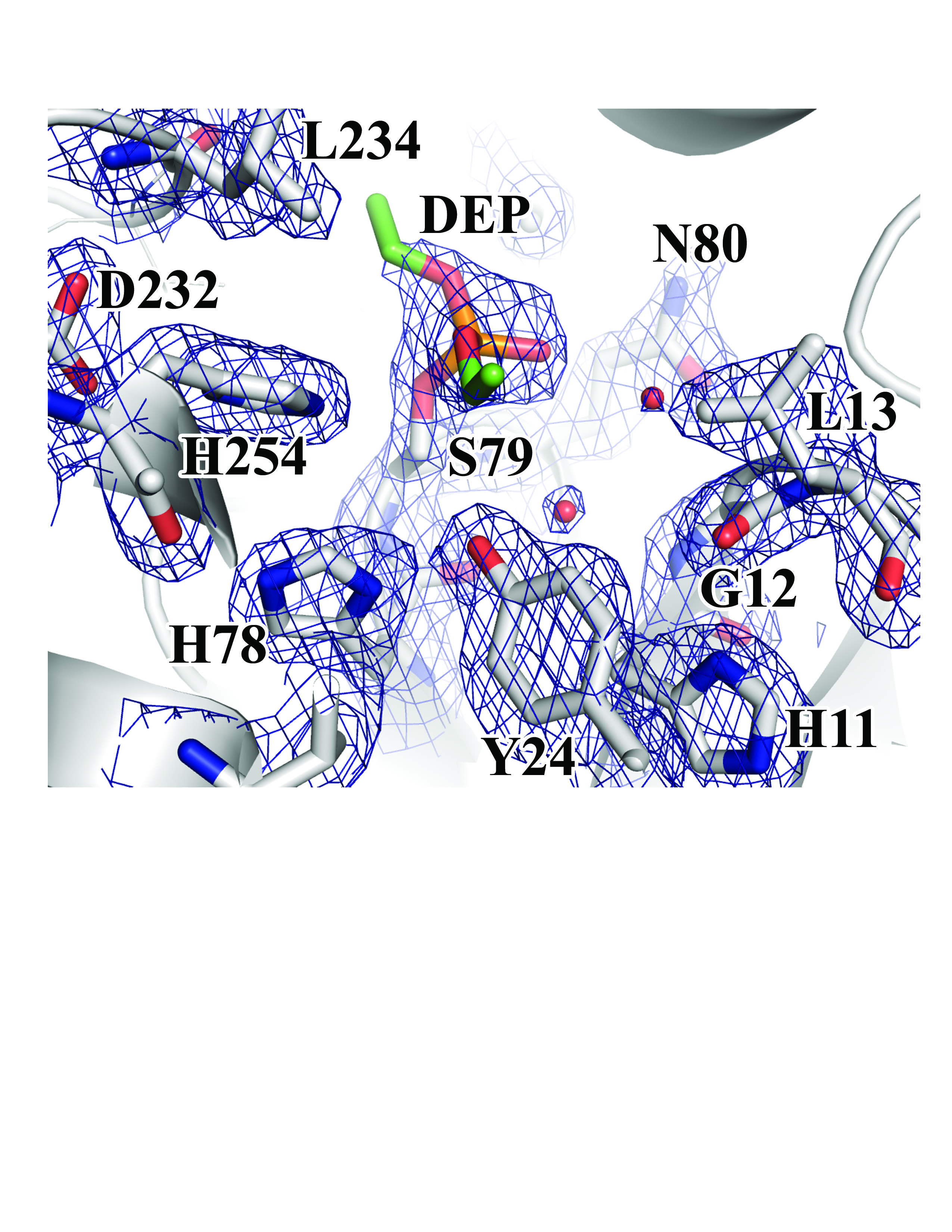

Supplement: Figure S1 — 2Fo-Fc sa-omit map contoured at 1 σ highlighting the covalently bound diethyl-phosphonate (DEP) inhibitor in the active site. (TIF) [file pone.0052890.s001.tif]

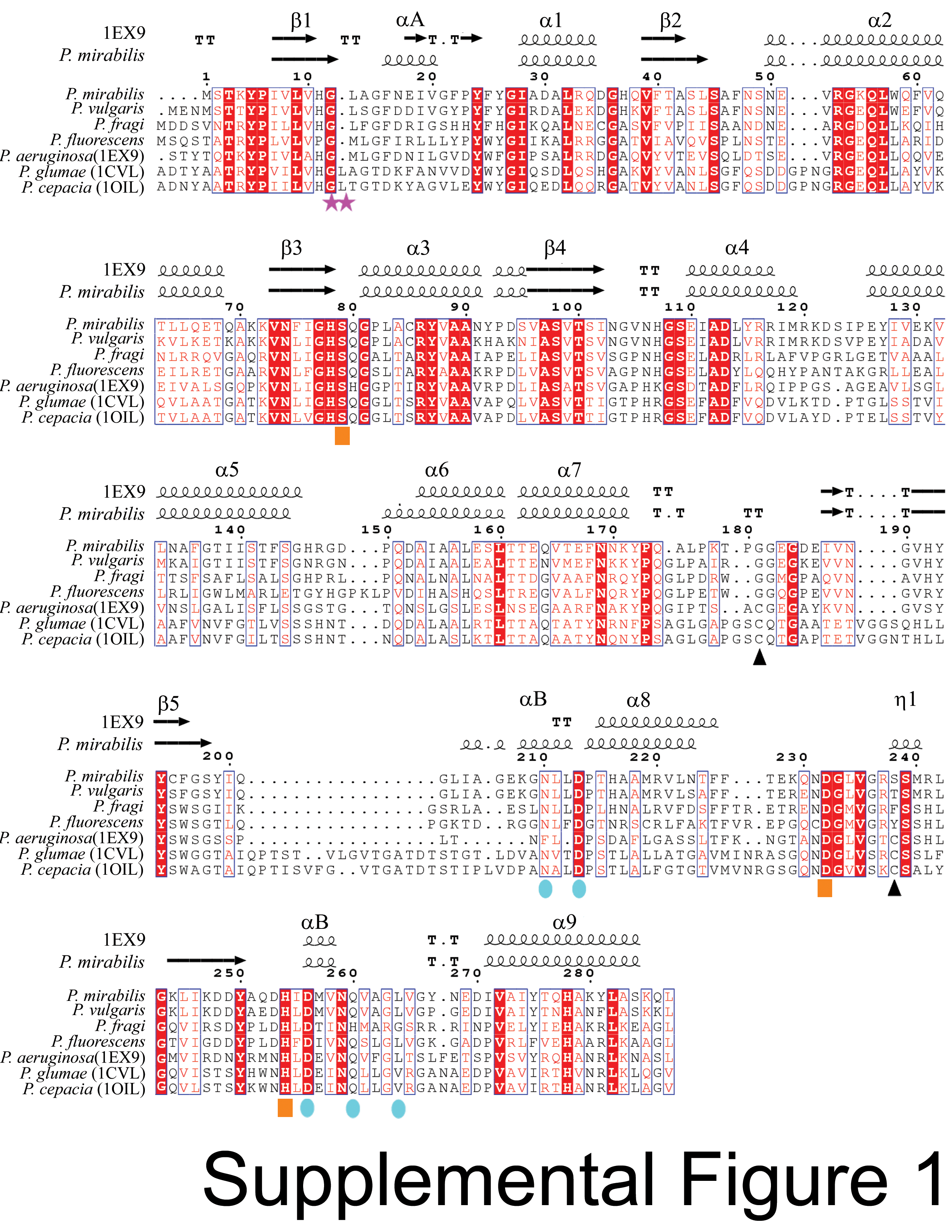

Supplement: Figure S2 — Multiple sequence alignment of psychrophilic and mesophilic lipases from lipase family I.1 and I.2. The secondary structure elements are numbered as in Figure 1. Orange squares, catalytic triad residues. Ca2+ binding site, blue ovals. Disulfide bond, black triangle. (TIF) [file pone.0052890.s002.tif]

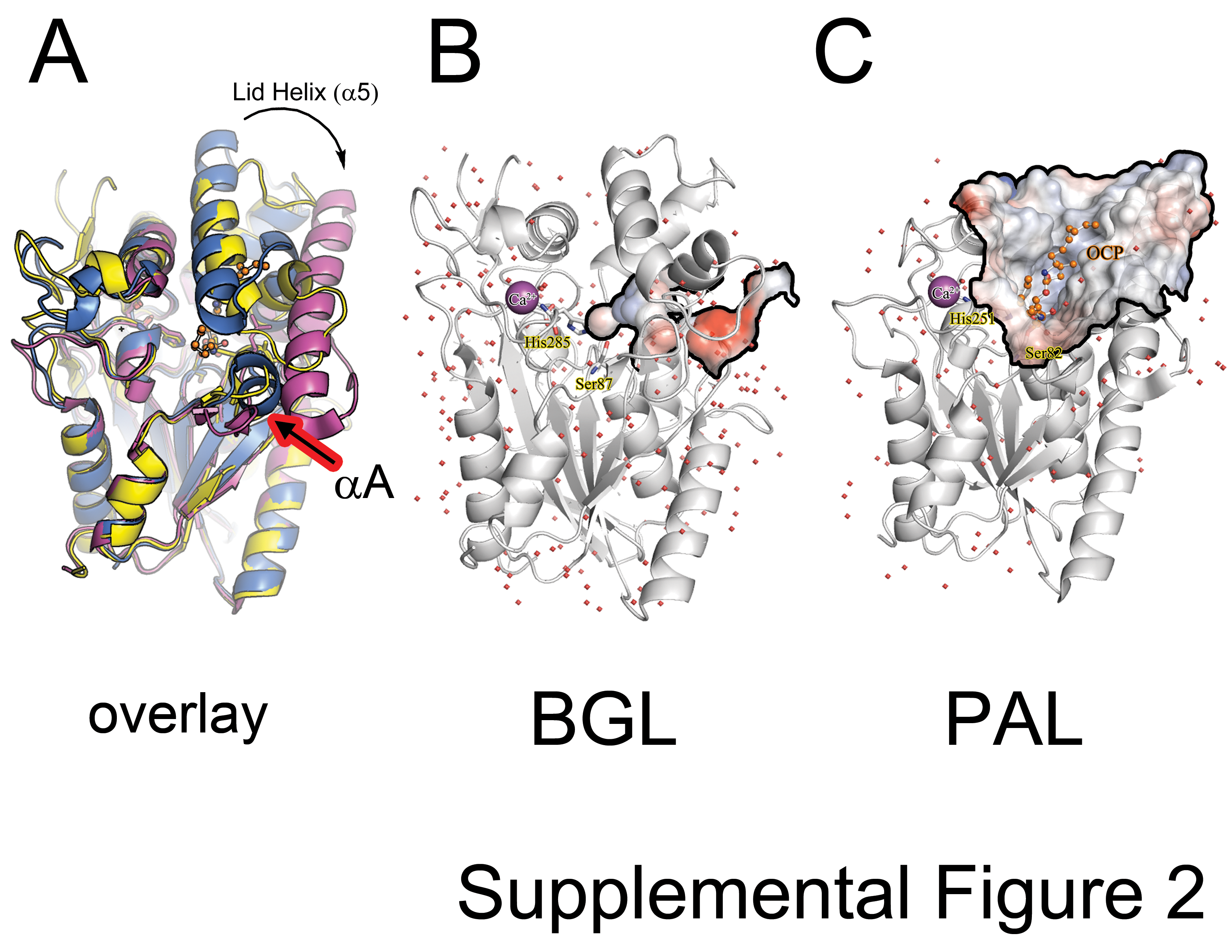

Supplement: Figure S3 — Comparison of the open and closed conformations seen in family I.1 and I.2 lipases. A) Overlay of closed PML (blue), closed B. glumae (PDBID:1CVL), and open P. aeruginosa (PDBID:1EX9) lipases. The helix αA that would need to move upon lid opening is highlighted with a red arrow. B) Surface representation of the solvent accessibility of the active site pocket of B. glumae (closed) and P. aeruginosa (open). The active site of B. glumae lipase is marginally accessible in the closed conformation. The open conformation of P. aeruginosa lipase shows a hydrophobic cleft that can accommodate a triglyceride analog (orange ball and stick) without steric clashes. (TIF) [file pone.0052890.s003.tif]
