# Supplementary material for: Crystal Structure of Proteus mirabilis Lipase, a Novel Lipase from the Proteus/Psychrophilic Subfamily of Lipase Family I.1
Source: PLoS One. 2012 Dec 26;7(12):e52890. doi: 10.1371/journal.pone.0052890 (PMC3530535; doi:10.1371/journal.pone.0052890)
Supplement: Table S1 — Crystallization, data collection and refinement statistics for the crystal structures of the apo and DEP inhibited P. mirabilis lipase. (DOC) [file pone.0052890.s004.doc]

|  | PML-Native | PML-DEP |
| --- | --- | --- |
| **A. Crystallization** | 0.095 M NaCitrate pH 5.6  19% IsoPropanol 19% PEG4000  5% glycerol | 0.1 M Bis-Tris Propane pH 6.5  16% PEG2000 MME |
| **B. Crystallographic Data** |  |  |
| Space Group | P3_2_ | P3_2_ |
| Cell Dimension (Å) | 65.44 65.44 63.97 α=β=90, γ=120 | 65.57, 65.57, 63.46 α=β=90, γ=120 |
| Resolution (Å) | 2.00 | 2.20 |
| No. of Unique Reflections | 20166 | 20659 |
| Completeness % (last shell) | 97.7 (96.2) | 99.8 (99.9) |
| I/σ(I) (last shell) | 10.02 (3.37) | 9.44 (3.05) |
| Redundancy | 3.8 (3.8) | 3.2 (3.1) |
| R_merge_ % (linear/square) | 13.7/12.2(43.0/41.3) | 10.8/9.7 (45.4/45.2) |
| **C. Refinement** |  |  |
| Resolution (Å) | 29.13-2.00 | 32.79-2.20 |
| No. Reflections (all/work) | 19181 (1467) | 13160 (916) |
| No. Protein Atoms | 2211 | 2211 |
| No. Ligand/Ion Atoms | 41 | 19 |
| No. Waters | 192 | 175 |
| Rfree % | 19.4 | 24.9 |
| Rcrys % | 16.7 | 19.5 |
| B-factor Protein (Å^2^) | 15.7 | 14.5 |
| B-factor Ligand/Ion (Å^2^) | 51.7 | 41.5 |
| B-factor Water (Å^2^) | 34.2 | 33.2 |
| **D. Geometry** |  |  |
| RMS Bonds (Å) | 0.004 | 0.005 |
| RMS Angles (deg°) | 0.871 | 0.872 |
| Ramachandran Plot (%) |  |  |
| Most Favored | 90.3 | 90.2 |
| Favored | 8.2 | 8.5 |
| Generously Allowed | 0.8 | 0.8 |
| Disallowed | 0.4 | 0.4 |
